# Bioactive Polysaccharides Prevent Lipopolysaccharide-Induced Intestinal Inflammation via Immunomodulation, Antioxidant Activity, and Microbiota Regulation

**DOI:** 10.3390/foods14152575

**Published:** 2025-07-23

**Authors:** Mingyang Gao, Wanqing Zhang, Yan Ma, Tingting Liu, Sijia Wang, Shuaihu Chen, Zhengli Wang, Hong Shen

**Affiliations:** College of Animal Science and Technology, Shihezi University, Shihezi 832000, China; gaomingyangnlbl@163.com (M.G.); zwq827222@163.com (W.Z.); myy0129@126.com (Y.M.); tingtingliu0212@163.com (T.L.); 0922@163.com (S.W.); 17867594001@163.com (S.C.); a843697356@outlook.com (Z.W.)

**Keywords:** gut microbiota, prebiotics, intestinal barrier, NF-κB/MyD88 signaling pathway, oxidative stress, IBD

## Abstract

Intestinal inflammation involves barrier impairment, immune hyperactivation, and oxidative stress imbalance. Bioactive polysaccharides universally alleviate inflammation via anti-inflammatory, antioxidant, and microbiota-modulating effects, yet exhibit distinct core mechanisms. Elucidating these differences is vital for targeted polysaccharide applications. This research examines distinct regulatory pathways through which diverse bioactive polysaccharides mitigate lipopolysaccharide-triggered intestinal inflammation in male Kunming (KM) mice. This experiment employed Lentinula edodes polysaccharide (LNT), Auricularia auricula polysaccharide (AAP), Cordyceps militaris polysaccharide (CMP), Lycium barbarum polysaccharide (LBP), and Brassica rapa polysaccharide (BRP). The expression levels of biomarkers associated with the TLR4 signaling pathway, oxidative stress, and intestinal barrier function were quantified, along with comprehensive gut microbiota profiling. The results showed that all five polysaccharides alleviated inflammatory responses in mice by inhibiting inflammatory cytokine release, reducing oxidative damage, and modulating gut microbiota, but their modes of action differed: LBP significantly suppressed the TLR-4/MyD88 signaling pathway and its downstream pro-inflammatory cytokine expression, thereby blocking inflammatory signal transduction and reducing oxidative damage; LNT and CMP enhanced the body’s antioxidant capacity by increasing antioxidant enzyme activities and decreasing malondialdehyde (MDA) levels; AAP and BRP enriched *Akkermansia* (Akk.) within the *Verrucomicrobia* (Ver.) phylum, upregulating tight junction protein expression to strengthen the intestinal mucosal barrier and indirectly reduce oxidative damage. This research demonstrates that different polysaccharides alleviate inflammation through multi-target synergistic mechanisms: LBP primarily inhibits inflammatory pathways; AAP and BRP focus on intestinal barrier protection and microbiota modulation; and LNT and CMP exert effects via antioxidant enzyme activation. These data support designing polysaccharide blends that leverage complementary inflammatory modulation mechanisms.

## 1. Introduction

Globally, inflammatory bowel disease (IBD) poses a considerable health burden, characterized by persistent and chronic intestinal inflammation [1]. IBD currently affects over 6.8 million individuals worldwide, with industrialized nations demonstrating particularly high disease burdens. Epidemiological studies have established ultra-processed foods, including extruded snacks, deep-fried items, refined grains, and sugar-sweetened beverages, as independent risk factors for IBD pathogenesis [2,3]. Beyond enduring chronic abdominal pain and debilitating diarrhea, patients with severe IBD face life-threatening complications such as intestinal perforation and colorectal carcinoma [4,5]. Current therapeutic strategies, while offering symptomatic relief, often entail adverse effects and limited long-term efficacy [6]. This urgent unmet need has driven the exploration of natural bioactive compounds as safe alternatives for preventing and alleviating the pathogenesis of IBD, particularly those targeting immune responses, oxidative stress, and gut dysbiosis triggered by it.

IBD pathogenesis emerges from a complex, multifactorial interplay involving an intricate web of genetic susceptibility, environmental triggers, and immune dysregulation. Disrupted intestinal barrier function and aberrant inflammatory pathway activation constitute fundamental pathophysiological features. Lipopolysaccharide (LPS), a Gram-negative bacterial endotoxin, initiates TLR4-dependent signaling upon receptor binding. This cascade instigates MyD88-dependent NF-κB activation, driving dysregulated synthesis of pro-inflammatory cytokines—particularly TNF-α and IL-1β. These events collectively establish a vicious cycle of mucosal injury [7,8,9,10,11]. Fundamentally, gut microbiota composition orchestrates host immunity and intestinal barrier competence—a paradigm substantiated by substantial evidence [12,13,14]. IBD patients manifest substantial depletion of salutary bacterial taxa—notably *Firmicutes* and *Akkermansia*—renowned for maintaining gut health and immune homeostasis. Conversely, pathobionts such as *Proteobacteria* expand, potentially disrupting microbial equilibrium and propagating inflammation-driven pathogenesis [15,16].

Bioactive polysaccharides, widely distributed in medicinal plants and fungi, demonstrate multifaceted anti-IBD properties, including immunomodulation, antioxidant activity, and microbiota regulation [17]. Over millennia, Traditional Chinese Medicine (TCM) formulations containing bioactive polysaccharides as key active components have been utilized to alleviate and treat various inflammatory conditions, including IBD. Modern pharmacological research demonstrates that natural polysaccharides derived from diverse TCM sources offer significant advantages, including high safety profiles, excellent biocompatibility, and low production costs [18]. Functioning as prebiotics, these polymeric compounds remain undegraded in the monogastric small intestine, yet are metabolized by diverse gut bacteria. Such modulation shifts microbial composition toward beneficial species while suppressing pathogens, thereby stabilizing the intestinal ecosystem. Furthermore, substantial research has confirmed that polysaccharides derived from traditional Chinese medicine can effectively alleviate inflammatory responses induced by IBD in animals through multiple pathways, including immunomodulation, restoration of gut microbial balance, and modulation of metabolic pathways such as NLRP3 inflammasome activation and AMPK/mTOR regulation [17,19,20,21,22]. LNT and CMP reduce the release of inflammatory cytokines [23,24], while LBP suppresses TLR4/MyD88 pathway activation and pro-inflammatory cytokine secretion while upregulating tight junction protein expression [25]. AAP maintains intestinal integrity by upregulating MUC2, ZO-1, Occludin, and Claudin expression [26], and BRP also exhibits protective effects against intestinal barrier damage [27]. However, systematic comparative research on the mechanistic differences among distinct bioactive polysaccharides remains scarce, and supporting evidence for combination polysaccharide strategies based on complementary mechanisms is also relatively lacking. This experiment selected KM male mice as the experimental animals and utilized the bioactive polysaccharides LNT, AAP, CMP, LBP, and BRP. Within TCM frameworks, these five polysaccharides function as bioactive agents exhibiting dual capacities: anti-inflammatory activity and gastrointestinal immune regulation [28,29]. Experimental studies have further validated their efficacy in alleviating IBD primarily through three core mechanisms: anti-inflammation, antioxidant stress response, and microbiota regulation [25,26,28,30,31]. Building upon established evidence that 200 mg/kg BW of these polysaccharides attenuates murine intestinal inflammation and restructures gut microbiota profiles [27,32,33,34,35], we implemented consistent dosing via daily intragastric gavage to ensure translational validity.

We hypothesize that five clinically significant polysaccharides all possess abilities to inhibit the TLR4 pathway, activate antioxidant enzymes, and modulate the microbial barrier function. However, the extent to which they regulate these different mechanisms varies, leading to divergent effects in restoring barrier integrity and mitigating LPS-induced intestinal inflammation to varying degrees. We systematically compare five polysaccharides’ efficacy in alleviating murine LPS-colitis, integrating analyses of TLR4/MyD88/NF-κB activity, oxidative stress, gut microbiota, and barrier integrity to reveal differential mechanisms. This provides a foundation for anti-inflammatory polysaccharide blend strategies based on complementary regulatory actions.

## 2. Materials and Methods

### 2.1. Experimental Materials

LNT, AAP, CMP, and LBP (≥95% purity) were commercially acquired from Shanghai Huzhen Biotechnology Co. (Shanghai, China). BRP (purity ≥ 95%) and LPS (≥97% purity) were respectively procured from Shaanxi Hanna Biotechnology Co. (Xi’an, China) and Sigma-Aldrich (Saint Louis, MO, USA).

### 2.2. Experimental Design

This study was conducted with approval from Shihezi University’s Animal Ethics Committee (Authorization No. A2025-545). The experiment was carried out at the Animal Center of Shihezi University, where extensive facilities and resources were utilized to ensure the precision and reliability of the research endeavors. Throughout this study, we strictly adhered to internationally recognized principles of animal welfare and ethics. This included providing animals with clean living environments meeting standard requirements, sufficient food and water, and implementing measures to minimize animal suffering.

KM mice have found extensive and diverse applications in the fields of drug testing and disease simulation. Their utility in these areas has been well-documented and recognized. In total, 420 robust male KM mice, which were 5 weeks old, of SPF grade, and had an average weight of 27.38 ± 1.5 g, were obtained from the Xinjiang Medical University Experimental Animal Center. These mice resided in a carefully regulated SPF environment that maintained an optimal temperature of 22 ± 2 °C, a relative humidity range of 55% to 60%, and a 12-h light/dark cycle to ensure their well-being and consistency in experimental results After a thorough and meticulous 10-day adjustment phase, which allowed the KM mice ample opportunity to freely access both food and water in order to fully acclimate and adapt to their novel surroundings, they subsequently underwent a randomized division process, being allocated into seven distinct and separate groups, with this allocation being based specifically on mice body weight measurements. These groups included the Control group (CON), the LPS group, the LPS+LNT group, the LPS+AAP group, the LPS+CMP group, the LPS+LBP group, and the LPS+BRP group. Every group was composed of 60 mice, each replicated 6 times, with 10 mice in each replication. Throughout the experimental period, each group received a daily oral dose of their specific polysaccharide solutions at a concentration of 200 mg/kg body weight for a consecutive span of 10 days at predetermined intervals. The CON and LPS groups received a similar amount of saline instead of the polysaccharide solutions as a control measure. On the 11th day of the experiment, each mouse was administered an intraperitoneal injection of LPS at a dose of 5 mg/kg body weight to induce the desired physiological response. Throughout the duration of this study, all mice were granted unrestricted access to both water and food to minimize any potential stressors that could affect the results. The disease activity index (DAI) was assessed through daily documentation of body weight loss, stool consistency, and severe rectal bleeding [36]. Six hours after the injection process was completed, six mice from each group were meticulously selected based on their DAI scores. These mice were then subjected to cervical dislocation in a humane manner. Subsequent to euthanasia, a comprehensive collection of samples was initiated, encompassing blood samples for hematological analysis, tissue samples for histological examination, and fecal samples for microbiological and biochemical assessments. These collections were meticulously documented and stored under appropriate conditions to facilitate subsequent detailed analysis.

### 2.3. Immune Organ Indices

Murine spleen, thymus, and liver tissues were harvested, subjected to sterile saline rinsing, and weighed for immune organ index computation using standardized formulae:

Spleen index (mg/g) = Spleen weight (mg)/Body weight (g)(1)

Thymus index (mg/g) = Thymus weight (mg)/Body weight (g)(2)

Liver index (mg/g) = Liver weight (mg)/Body weight (g)(3)

### 2.4. Antioxidant Capacity Assessment

Following thawing at 4 °C, jejunal tissues were saline-rinsed, homogenized in 0.45 mL of ice-cold PBS, and centrifuged at 8000× *g* (10 min, 4 °C). The resultant supernatants underwent ELISA analysis (Servicebio, Wuhan, China) to quantify antioxidant markers according to standardized protocols.

### 2.5. Immune Parameter Analysis

Plasma and jejunal tissues were thawed at 4 °C. Post-thawing, jejunal specimens underwent sterile saline rinsing, homogenization in 0.45 mL of ice-cold PBS, and centrifugation (8000× *g*, 10 min, 4 °C). The resultant supernatants were assayed for immunological parameters using commercial ELISA kits (Servicebio, Wuhan, China) following manufacturer protocols.

### 2.6. Histological Characterization of Intestinal Tissues

Segments of the intestine were preserved in 4% paraformaldehyde (PFA) for a day under light protection, then embedded in paraffin and sectioned into 3 μm thick slices. For histological evaluation, hematoxylin and eosin (H&E) staining was performed. The process of measuring villus height and crypt depth involved the utilization of a light microscope (BX53, Olympus Corporation, Tokyo, Japan).

### 2.7. Immune and Tight Junction-Related Gene Expression

To isolate total RNA, jejunal tissues were homogenized with the TransZoL Up Plus Kit. The extracted RNA was then reverse-transcribed into cDNA using BioGold Q RT SuperMix. The levels of TLR4, MyD88, TNF-α, NF-κB, and tight junction proteins (occludin, ZO-1, MUC2) in mRNA were measured using RT-qPCR and gene-specific primers (refer to Table 1), all produced by Xinjiang Youkang Biotechnology Co. (Ürümqi, China). β-actin was used as an internal control, and its relative expression was determined through the 2−ΔΔCt technique.

### 2.8. Analysis of Gut Microbiota Composition

Shanghai Majorbio Bio-Pharm Technology Co. (Shanghai, China) conducted 16S rDNA sequencing. Cecal content samples were transferred to their facility for total DNA extraction. Genomic DNA integrity was confirmed via 1% agarose gel electrophoresis. PCR amplification targeted the V3–V4 hypervariable regions of bacterial 16S rRNA using universal primers 338F (5′-ACTCCTACGGGAGGCAGCAG-3′) and 806R (5′-GGACTACHVGGGTWTCTAAT-3′).

### 2.9. Statistical Analysis and Data Visualization

Statistical analyses utilized SPSS 27 (IBM, New York, NY, USA) with dataset normality verified by the Shapiro–Wilk test (*p* > 0.05). Intergroup differences were assessed via one-way ANOVA and LSD post hoc testing. Data visualization employed GraphPad Prism 10.1.2, while correlation and clustering analyses used Origin 2021. The clustering analysis employed hierarchical clustering. Investigators remained blinded to group assignments during data collection and analysis.

## 3. Results

### 3.1. Relief of Inflammatory Symptoms by Five Polysaccharides

**Figure 1:**
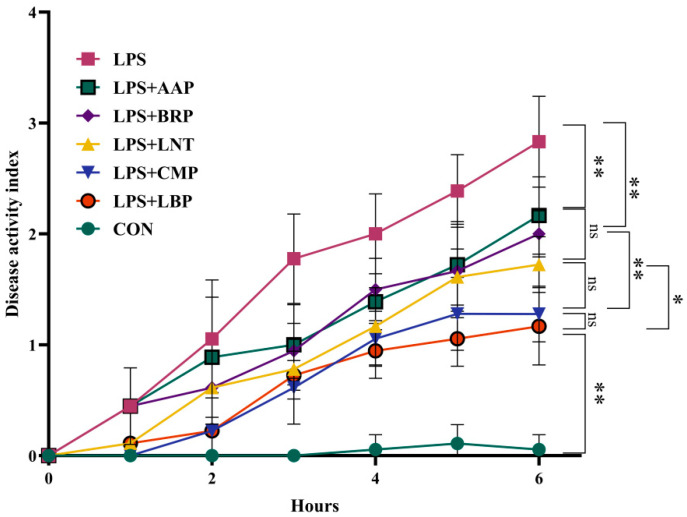
The influence of polysaccharides on the disease activity index. (*n* = 6). * *p* < 0.05, ** *p* < 0.01, ^ns^ *p* < 0.05.

Figure 1 indicates that, at 1 hour post-induction, DAI values in LPS+LNT, LPS+LBP, and LPS+CMP groups showed no statistical divergence from CON controls. By 6 h, all polysaccharide-treated cohorts manifested significantly reduced DAI scores versus the LPS group (*p* < 0.01). Among interventions, LPS+LBP demonstrated lower DAI than LPS+LNT (*p* < 0.05). Furthermore, LPS+LBP and LPS+CMP groups exhibited decreased DAI relative to LPS+BRP and LPS+AAP groups (*p* < 0.01).

### 3.2. Effects of Five Polysaccharides on Immune Organ Indices

Table 2 delineates a significant reduction in thymic index in the LPS group versus CON controls (*p* < 0.05). Conversely, splenic indices increased in LPS+LNT, LPS+AAP, LPS+LBP, and LPS+BRP cohorts relative to both CON and LPS groups (*p* < 0.05).

### 3.3. Effects of Five Polysaccharides on Antioxidant Parameters

**Figure 2:**
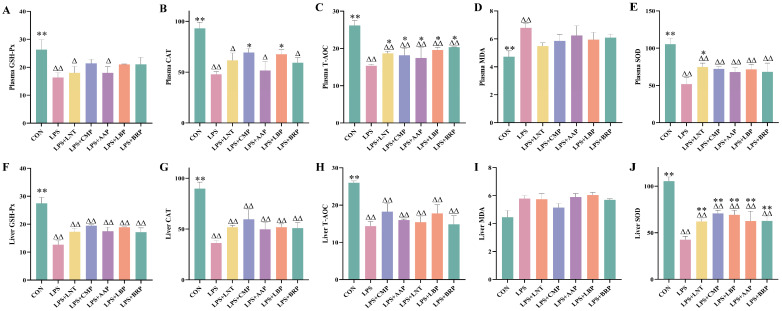
Effects of polysaccharides on antioxidant indicators (*n* = 6). (**A**) Plasma GSH-Px, (**B**) Plasma CAT, (**C**) Plasma T-AOC, (**D**) Plasma MDA, (**E**) Plasma SOD, (**F**) Liver GSH-Px, (**G**) Liver CAT, (**H**) Liver T-AOC, (**I**) Liver MDA, and (**J**) Liver SOD. ^∆^ *p* < 0.05, ^∆∆^ *p* < 0.01, relative to the CON group. * *p* < 0.05, ** *p* < 0.01, relative to the LPS group.

As summarized in Figure 2, the LPS group exhibited reductions in the GSH-Px content, CAT activity, SOD activity, and T-AOC in both plasma and liver tissues (*p* < 0.01), accompanied by an elevated MDA content in plasma (*p* < 0.01). Relative to the LPS group, the LPS+LNT group exhibited increased plasma T-AOC and hepatic/plasma SOD activity (*p* < 0.01). Similarly, the LPS+CMP and LPS+LBP groups exhibited elevated plasma CAT activity, T-AOC, and SOD activity (*p* < 0.05), accompanied by enhancements in hepatic SOD activity (*p* < 0.01). Notably, the LPS+CMP group demonstrated a significant decrease in the plasma MDA content (*p* < 0.05). Furthermore, both the LPS+AAP and LPS+BRP groups displayed elevations in plasma T-AOC and SOD activity (*p* < 0.05), with upregulation of hepatic SOD activity (*p* < 0.01).

### 3.4. Effects of Five Polysaccharides on the Levels of Immune-Related Cytokines and Inflammatory Factors

**Figure 3:**
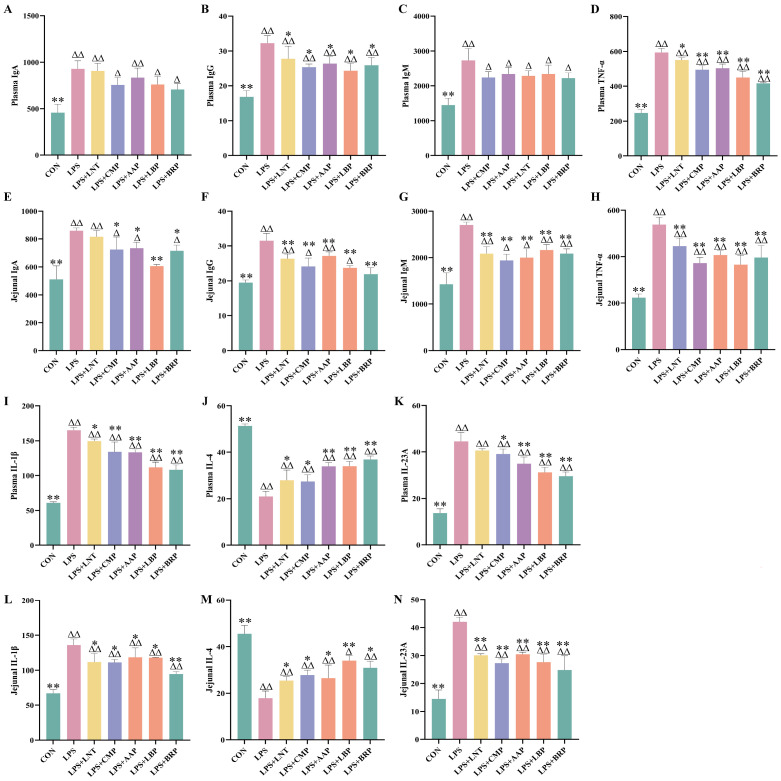
Effects of polysaccharides on immune indicators. (*n* = 6). (**A**) Plasma IgA, (**B**) Plasma IgA, (**C**) Plasma IgM, (**D**) Plasma TNF-α, (**E**) Jejunal IgA, (**F**) Jejunal IgA, (**G**) Jejunal IgM, (**H**) Jejunal TNF-α, (**I**) Plasma IL-1β, (**J**) Plasma IL-4, (**K**) Plasma IL-23A, (**L**) Jejunal IL-1β, (**M**) Jejunal IL-4, and (**N**) Jejunal IL-23A.^∆^ *p* < 0.05, ^∆∆^ *p* < 0.01, relative to the CON group. * *p* < 0.05, ** *p* < 0.01, relative to the LPS group.

Figure 3 demonstrates that LPS challenge significantly elevated pro-inflammatory mediators (TNF-α, IL-1β, and IL-23A) and immunoglobulins (IgA/IgG/IgM) in plasma and jejunum versus CON (*p* < 0.01), concomitant with IL-4 suppression (*p* < 0.01). All polysaccharide interventions reversed these effects: LPS+LNT suppressed plasma IgG/TNF-α/IL-1β (*p* < 0.05) and jejunal IgG/IgM/TNF-α/IL-23A (*p* < 0.01) while upregulating IL-4 (*p* < 0.05); LPS+CMP reduced plasma IgG/IL-23A and jejunal IgA/IL-1β (*p* < 0.05), suppressing plasma TNF-α/IL-1β and jejunal IgG/IgM/TNF-α/IL-23A (*p* < 0.01); LPS+AAP decreased plasma IgG/jejunal IgA (*p* < 0.05), inhibited TNF-α/IL-1β/IL-23A systemically (*p* < 0.01), and elevated IL-4 (*p* < 0.05); LPS+LBP decreased plasma TNF-α/IL-1β/IL-23A and jejunal IgA/IgG/IgM/TNF-α/IL-23A (*p* < 0.01) with a concurrent IL-4 increase (*p* < 0.01); LPS+BRP decreased plasma IgG/jejunal IgA (*p* < 0.05) and pro-inflammatory cytokines (*p* < 0.01), while upregulating IL-4 (*p* < 0.05).

### 3.5. Effects of Five Polysaccharides on TLR4/NF-κB Signaling Pathway-Associated Gene Expression

**Figure 4:**
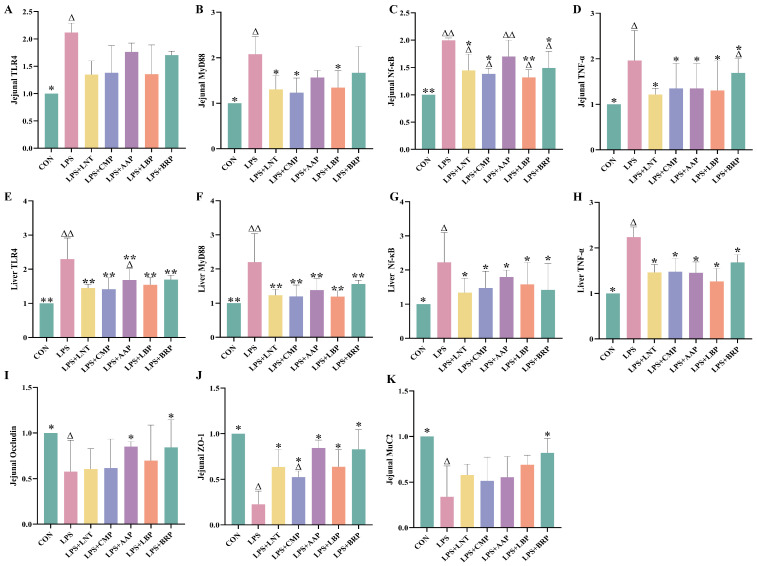
Effect of polysaccharides on the expression of immune and intestinal barrier-related genes (*n* = 6). (**A**) Jejunal TLR4, (**B**) Jejunal MyD88, (**C**) Jejunal Nf-κB, (**D**) Jejunal TNF-α, (**E**) Liver TLR4, (**F**) Liver MyD88, (**G**) Liver Nf-κB, (**H**) Liver TNF-α, (**I**) Jejunal Occludin, (**J**) Jejunal ZO-1, and (**K**) Jejunal MuC2. ^∆^ *p* < 0.05, ^∆∆^ *p* < 0.01, relative to the CON group. * *p* < 0.05, ** *p* < 0.01, relative to the LPS group.

Figure 4A–H demonstrates significant upregulation of TLR4/NF-κB pathway-associated genes in jejunal and hepatic tissues of the LPS group versus CON controls (*p* < 0.05). All polysaccharide interventions reduced plasma TNF-α mRNA expression relative to LPS (*p* < 0.05). Specifically, LPS+LBP, LPS+LNT, and LPS+CMP attenuated LPS-induced elevations in plasma MyD88 and NF-κB mRNA (*p* < 0.05), with LPS+LBP exhibiting particularly potent NF-κB mRNA suppression (*p* < 0.01). All polysaccharide-treated cohorts additionally downregulated hepatic TLR4/NF-κB pathway gene expression (*p* < 0.05).

### 3.6. Effects of Five Polysaccharides on the Expression of Genes Related to Jejunal Intestinal Barrier

Figure 4I–K reveals diminished jejunal transcript abundance of occludin, ZO-1, and MUC2 in LPS-challenged mice (*p* < 0.05). The LPS+BRP group exhibited upregulation of occludin and MUC2 mRNA compared with the LPS group. (*p* < 0.05), while the LPS+AAP group displayed elevated occludin mRNA levels (*p* < 0.05). All polysaccharide-treated groups demonstrated increases in ZO-1 mRNA expression (*p* < 0.05).

### 3.7. Effects of Five Polysaccharides on Jejunal Tissue Morphology

As demonstrated in Table 3, the LPS group exhibited decreased villous length and crypt depth across all small intestinal segments when compared with the control group (*p* < 0.01). All polysaccharide interventions alleviated LPS-induced intestinal morphological damage (*p* < 0.05). Notably, the LPS+BRP group exhibited attenuation of villous and crypt atrophy across all intestinal segments (*p* < 0.01). The LPS+LNT, LPS+AAP, and LPS+LBP groups demonstrated a significant attenuation of villous and crypt atrophy in the duodenum, jejunum, and ileum (*p* < 0.01). Likewise, the LPS+CMP group exhibited restoration of villous length across all segments and crypt depth in the duodenum and ileum (*p* < 0.01).

### 3.8. Analysis of Diversity of Cecal Flora by Five Polysaccharides

**Figure 5:**
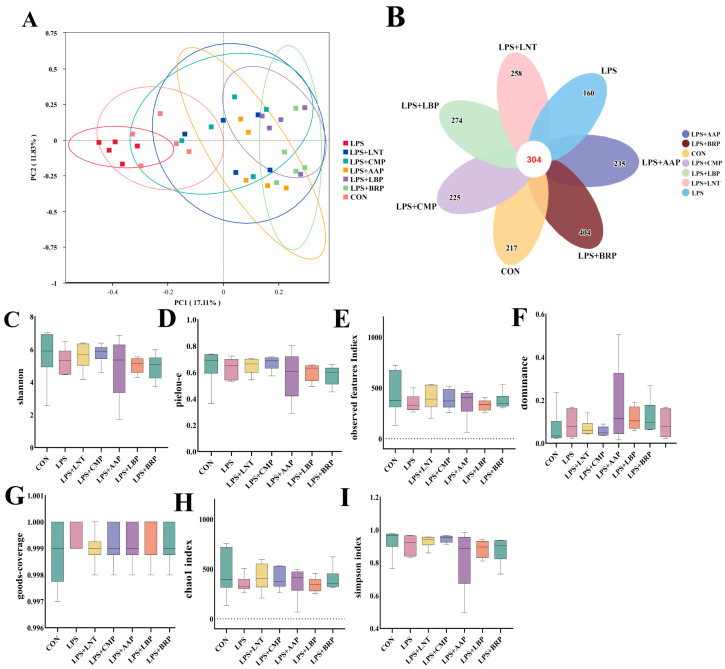
The effects of different polysaccharides on the species diversity of the intestinal flora (*n* = 6). (**A**) PCoA plot, (**B**) Petal plot presents each group of OTUs, (**C**) shannon, (**D**) pielou_e, (**E**) observed features, (**F**) dominance, (**G**) good_coverage, (**H**) chao1, (**I**) simpson.

Figure 5 reveals a pan-group taxonomic inventory of 2077 OTUs, with 304 conserved operational units constituting the microbial core. The LPS group harbored the lowest number of unique OTUs (*n* = 160), whereas the LPS+BRP group showed the highest unique OTU count (*n* = 404), exceeding that of other groups (*p* < 0.05) (Figure 5B). PCoA utilizing Bray–Curtis distances demonstrated distinct clustering patterns in the LPS group, in contrast to the LPS+AAP, LPS+BRP, and LPS+LBP groups (Figure 5A). Compared with the LPS group, all polysaccharide-treated groups exhibited increases in Observed_species, Simpson, and Chao1 indices (*p* > 0.05). This trend suggests a potential enhancement in species richness, diversity, and community evenness of the gut microbiota (Figure 5C–I).

### 3.9. Effect of Five Polysaccharides on the Microbial Composition of Mouse Cecum

**Figure 6:**
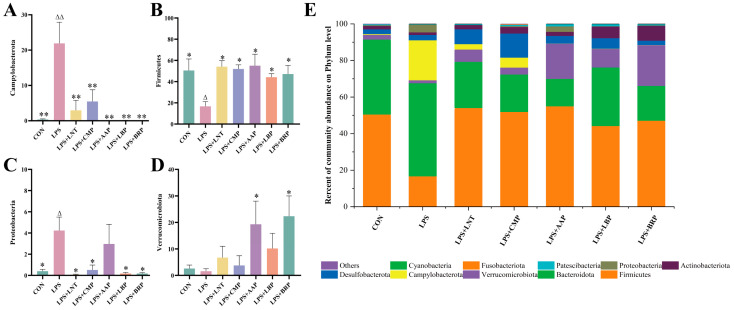
Effects of various polysaccharides on the intestinal microbiota at the phylum level (*n* = 6): (**A**) *Campylobacterota*, (**B**) *Firmicutes*, (**C**) *Proteobacteria*, and (**D**) *Verrucomicrobiota*, and (**E**) The role of polysaccharides in regulating the structure of intestinal microbiota at the phylum level. ^∆^ *p* < 0.05, ^∆∆^ *p* < 0.01, relative to the CON group. * *p* < 0.05, ** *p* < 0.01, relative to the LPS group.

In terms of phylum hierarchy, the LPS group had the highest number of *Fungi*, *Bacteroidetes*, and *Campylobacter phyla*, whereas in the other groups, *Fungi*, *Bacteroidetes*, and *Verrucomicrobium phyla* dominated (Figure 6E). The LPS group exhibited a significant increase in *Campylobacter* abundance compared with the CON group (*p* < 0.01), as well as elevations in *Proteobacteria* (*p* < 0.05), but a reduction in *Firmicutes* (*p* < 0.05). In contrast, all polysaccharide groups demonstrated decreases in *Campylobacterota* (*p* < 0.01) and *Proteobacteria* (*p* < 0.05) relative to the LPS group. With the exception of the LPS+AAP group, all polysaccharide interventions led to a reduction in *Firmicutes* abundance (*p* < 0.05). Notably, the LPS+AAP and LPS+BRP groups exhibited increases in *Verrucomicrobiota* (*p* < 0.05).

**Figure 7:**
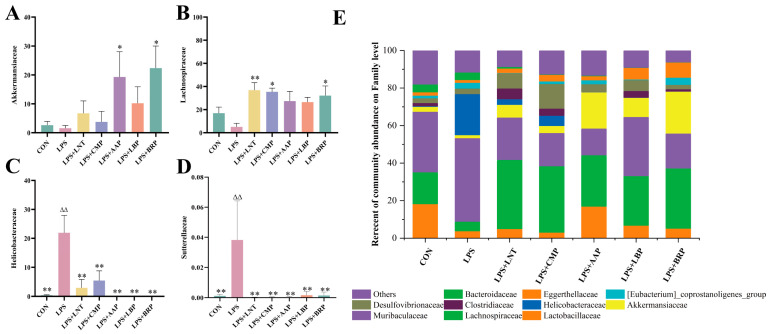
Effects of various polysaccharides on the intestinal microbiota at the family level (*n* = 6): (**A**) *Akkermansiaceae*, (**B**) *Lachnospiraceae*, (**C**) *Helicobacteraceae*, and (**D**) *Sutterellaceae*, and (**E**) The role of polysaccharides in regulating the structure of intestinal flora at the genus level. ^∆∆^ *p* < 0.01, relative to the CON group. * *p* < 0.05, ** *p* < 0.01, relative to the LPS group.

At the family level, Muribaculaceae prevailed in the CON, LPS, and LPS+LBP cohorts, whereas Lachnospiraceae dominated the LPS+LNT, LPS+CMP, LPS+AAP, and LPS+BRP groups (Figure 7E). Compared with LPS, LPS+LNT elevated Helicobacteriaceae and Sutterellaceae abundance (*p* < 0.01). Elevations in Lachnospiraceae occurred in LPS+CMP/BRP groups (*p* < 0.05), while LPS+AAP/BRP augmented Akkermansiaceae (*p* < 0.05). All polysaccharide interventions reduced Helicobacteraceae versus LPS (*p* < 0.01) (Figure 7A–D).

**Figure 8:**
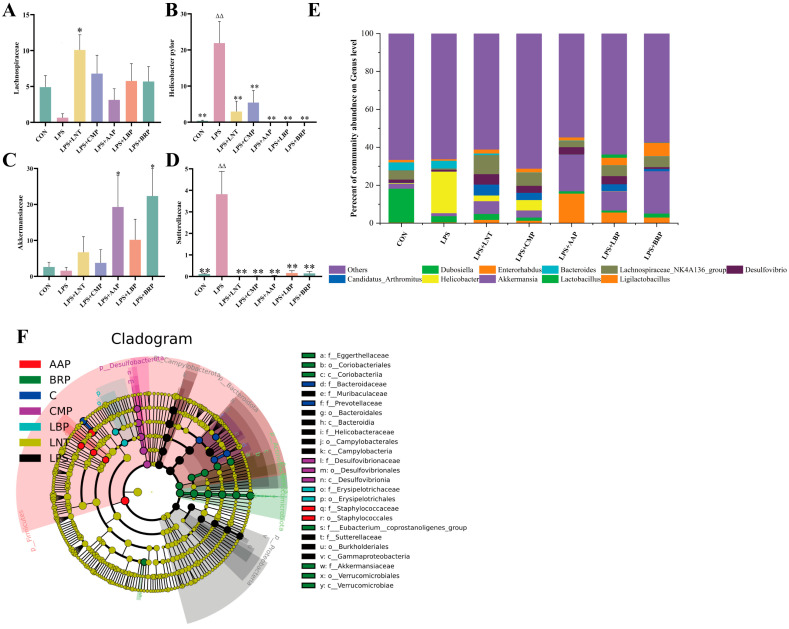
Effects of various polysaccharides on the intestinal microbiota at the genus level (*n* = 6): (**A**) *Lachnospiraceae*, (**B**) *Helicobacteraceaei*, (**C**) *Akkermansia*, and (**D**) *Sutterellaceae*; (**E**) The role of polysaccharides in regulating the genus-level structure of intestinal microbiota; and (**F**) LEfSe analysis. ^∆∆^ *p* < 0.01, relative to the CON group. * *p* < 0.05, ** *p* < 0.01, relative to the LPS group.

At the genus level, Helicobacter predominated in the LPS group, contrasting with the dominance of Lactobacillus in CON controls. Akkermansia prevailed in LPS+AAP/LBP/BRP groups, while Lachnospiraceae dominated LNT/CMP cohorts (Figure 8E). Versus CON, LPS significantly increased the abundance of Helicobacteraceae and Sutterellaceae (*p* < 0.01). LNT elevated Lachnospiraceae (*p* < 0.05), whereas LPS+AAP/BRP groups augmented Akkermansia levels (*p* < 0.05) (Figure 8A–D).

LEfSe analysis with LDA scores (threshold = 4) identified 15 potential microbial biomarkers in the LPS group, 6 in the CON group, 10 in the LPS+LNT group, 12 in the LPS+LBP group, 5 in the LPS+CMP group, 6 in the LPS+AAP group, and 2 in the LPS+BRP group (Figure 8F).

### 3.10. Correlation Analysis

**Figure 9:**
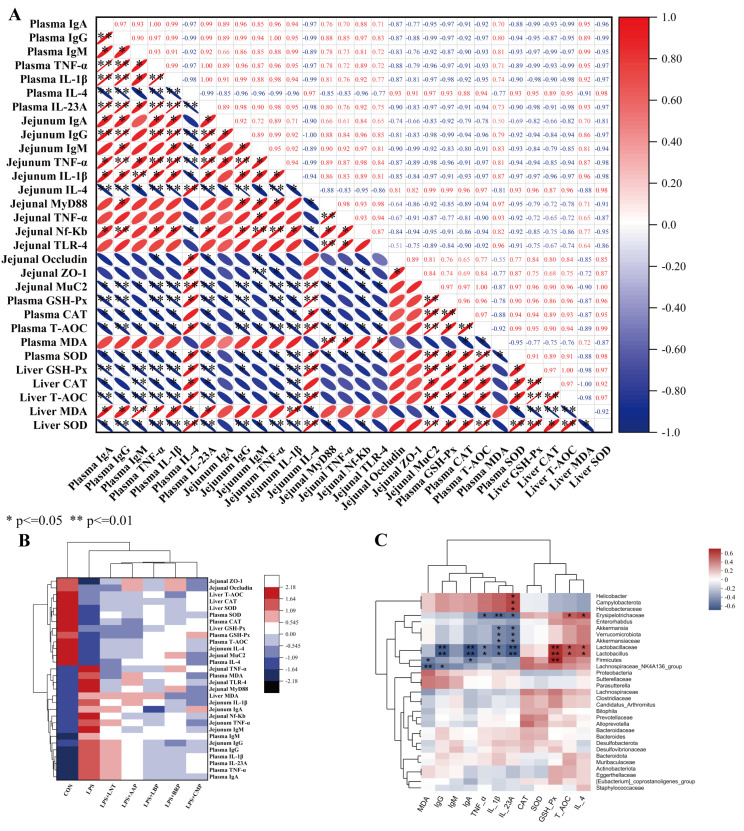
Heat map of the results of correlation analysis: (**A**) Correlation analysis of inflammatory factors, antioxidants, and intestinal barrier related indicators; (**B**) Inter-group cluster analysis of inflammatory factors, antioxidants, and intestinal barrier-related indicators; (**C**) Cluster analysis of plasma inflammatory factors, antioxidant indicators, and intestinal flora. Red signifies positive correlation, with intensity proportional to depth (deeper red indicating stronger positive correlation); blue denotes negative correlation, with intensity similarly proportional to depth (deeper blue indicating stronger negative correlation). * *p* < 0.05, ** *p* < 0.01.

Figure 9A illustrates the correlations among immune/inflammatory cytokines, oxidative stress markers, and intestinal tight-junction proteins across experimental groups (r < −0.7, *p* < 0.05), but positive correlations with anti-inflammatory cytokine levels (r > 0.7, *p* < 0.05). Figure 9B delineates the CON-to-LPS transition featuring TLR4/NF-κB cascade amplification and downstream pro-inflammatory effector induction, concurrent with the attenuation of anti-inflammatory signals, redox biomarkers, and intestinal barrier constituents. Phytoglycan administration universally mitigated these dysregulations.

Figure 9C delineates microbiota–immune/oxidative marker clustering. Phylum-level: Firmicutes abundance positively covaried with GSH-Px (r = 0.7, *p* < 0.01) but negatively covaried with IgA/MDA (r = −0.4, *p* < 0.05). Verrucomicrobiota was negatively correlated with IL-1β/IL-23A (r = −0.4, *p* < 0.05), while Campylobacterota showed a positive correlation with IL-23A (r = 0.4, *p* < 0.05). Family-level: Lactobacillaceae exhibited positive correlations with T-AOC/IL-4 (r = 0.4, *p* < 0.05) and GSH-Px (r = 0.7, *p* < 0.01), but negative correlations with IL-1β/TNF-α (r = −0.4, *p* < 0.05), IL-23A/IgA/IgM (r = −0.7, *p* < 0.01). Akkermansiaceae was correlated negatively with IL-1β/IL-23A (r = −0.4, *p* < 0.05). Erysipelotrichaceae showed positive links to T-AOC/IL-4 (r = 0.4, *p* < 0.05) but negative links to IL-23A/TNF-α (r = −0.4, *p* < 0.05) and IL-1β (r = −0.7, *p* < 0.01). Helicobacteraceae was positively associated with IL-23A (r = 0.4, *p* < 0.05). Genus-level: Lactobacillus mirrored family-level correlations. Lachnospiraceae_NK4A136_group displayed negative correlations with IgG (r = −0.4, *p* < 0.05) and MDA (r = −0.7, *p* < 0.01). Akkermansia was negatively associated with IL-1β/IL-23A (r = −0.4, *p* < 0.05), while Helicobacter was positively correlated with IL-23A (r = 0.4, *p* < 0.05).

## 4. Discussion

LPS triggers inflammatory responses in animals, resulting in hepatic, splenic, and thymic atrophy, weight loss, diarrhea, and rectal bleeding [37]. It further triggers substantially, leading to inflammatory cascades and oxidative damage [38,39]. As natural bioactive compounds, polysaccharides can suppress inflammatory cytokine secretion and alleviate inflammatory responses. Oral delivery of ginger polysaccharides (100 mg/kg) [40] and ginseng polysaccharides (200 mg/kg) [41] has been shown to markedly attenuate DAI in murine colitis models. Oral gavage of 100 mg/kg sulfated yam polysaccharides attenuates LPS-induced thymic and hepatic atrophy in mice [42]. Zhang et al. [43] documented that 500 mg/kg Artemisia argyi polysaccharides suppresses plasma and intestinal IL-1β/TNF-α concentrations while concurrently enhancing anti-inflammatory IL-10 in diarrheic mice. Cellular studies by Tomasz et al. [44] demonstrated that treatment with 50 μg/mL Trametes versicolor polysaccharides resulted in reduced cytokine release from immune cells. In this study, all five polysaccharides attenuated LPS-induced immune organ atrophy and DAI elevation suppressed immunoglobulin and pro-inflammatory cytokine secretion and enhanced anti-inflammatory cytokine production, consistent with the findings of Zhang et al. To explore polysaccharides’ anti-inflammatory mechanisms, we examined their effects on the TLR4/MyD88 pathway. LPS binding to TLR4 activates the MyD88/NF-κB cascade, leading to pro-inflammatory cytokine release and inflammatory responses [45]. Chen et al. [46] demonstrated that 600 mg/kg Morus alba polysaccharides inhibited LPS/TLR4 pathway activation of mice. In this study, all polysaccharide-treated cohorts exhibited downregulated expression of TLR4 pathway-associated genes in both jejunum and liver tissues. This indicates suppressed TLR4/MyD88 pathway activity mediating anti-inflammatory effects, thus attenuating cytokine release. These findings align with the conclusions reported by Chen et al.

In DSS-induced IBD models, polysaccharides from Lycium barbarum LBP, AAP, and CMP have been shown to suppress the NF-κB pathway, thereby inhibiting intestinal inflammation [34,47,48]. LBP likely prevents IBD through the modulation of macrophage polarization in the DSS model. Wang et al. [49] demonstrated that 200 mg/kg LBP administration ameliorates inflammatory bowel disease progression by modulating STAT1/STAT6 signaling pathways, thereby influencing macrophage polarization. Polysaccharides derive their anti-inflammatory and immunomodulatory capacities from inherent structural–functional properties. In this study, LBP exhibited superior regulatory effects on the TLR4/MyD88 pathway compared with other polysaccharides, likely due to its high arabinogalactan content. Zheng et al. [50] found that arabinogalactan reduces pro-inflammatory cytokine expression in LPS-treated cells. In poultry, dietary supplementation with arabinogalactan-rich polysaccharides enhances immune performance and maintains intestinal homeostasis in broilers [51]. Zhao et al. [52] reported that 100 mg/kg LBP-derived arabinogalactan decreases intestinal IL-1β and TNF-α expression by 31% and increases ZO-1 and occludin mRNA levels by 39% and 17%, respectively, in colitis mice. This highlights the role of arabinogalactan in LBP in enhancing immunoregulation. In contrast, within the DSS-induced IBD model, LBP demonstrated comparable efficacy in alleviating intestinal inflammation to the findings in our current study [34,53]. Polysaccharides’ actions on macrophages may constitute an underlying mechanism for their immunoregulatory effects. The mannose receptor (MR) is known to recognize specific carbohydrate structures [54]. Studies have demonstrated that active polysaccharides modulate MR in LPS-stimulated mouse peritoneal macrophages, suppressing phagocytosis while reducing the level of TNF-α [55]. Moreover, Toll-like receptor 2 (TLR2) and TLR4 serve as primary receptors for active polysaccharide ligands [56,57], both critically involved in inflammation and immune regulation. These findings align with the observed significant modulation of the TLR4 pathway in the current study.

During inflammatory responses, pro-inflammatory cytokines trigger massive reactive oxygen species (ROS) release, disrupting the equilibrium between oxidative free-radical reactions and lipid peroxidation. ROS induce lipid oxidative degradation, leading to MDA formation—a critical biomarker for evaluating systemic oxidative stress [58]. Our results revealed positive correlations between MDA levels and TLR4/MyD88 signaling pathway activity, as well as its downstream pro-inflammatory cytokines. Antioxidant enzymes form an endogenous defense system against oxidative stress. Catalyzing the dismutation of superoxide anions (O^2−^) to hydrogen peroxide, SOD initiates a detoxification cascade wherein CAT and GPx cooperatively reduce this cytotoxic intermediate to benign water and oxygen [39], thereby mitigating oxidative damage. T-AOC provides a comprehensive assessment of systemic antioxidant potential. LNT and CMP interventions significantly suppressed LPS-induced plasma MDA elevation, evidencing ROS-scavenging efficacy. This resonates with established antioxidant profiles of botanical polysaccharides, exemplified by Camellia flower isolates [59], Abrus cantoniensis polysaccharides [22], and licorice polysaccharides [60], all of which reduce MDA levels. The modulation of the nuclear factor erythroid 2-related factor 2 (NRF2) pathway by polysaccharides potentially underpins their redox–regulatory competence, positioning this axis as a cardinal determinant of cytoprotective efficacy. As the master regulator of cellular antioxidant responses, the NRF2 pathway coordinates NRF2 protein phosphorylation and nuclear translocation upon oxidative stress. This promotes antioxidant response element (ARE) docking, initiating cascade activation of phase II detoxifying enzymes [61,62,63]. Fan et al. [64] demonstrated that bioactive polysaccharides enhance antioxidant capacity by activating the PI3K/AKT pathway, which thereby activates the NRF2 pathway in IPEC-J2 cells. This cascade upregulates antioxidant enzyme genes and mitigates oxidative stress, consistent with our experimental findings. Crucially, Nrf2 knockout models completely abolished the protective effects of LBP [65], definitively establishing the NRF2 pathway as the pivotal mechanistic axis through which bioactive polysaccharides confer resistance to oxidative stress. Despite universal enhancement of plasma T-AOC across polysaccharide interventions, their antioxidant enzyme induction profiles diverged markedly: LNT preferentially boosted GSH-Px/SOD activities, contrasting with CAT-specific potentiation by CMP and LBP. These findings unveil compound-specific redox regulation architectures. LNT primarily consists of pyranose rings linked by β-glycosidic bonds. This structural configuration may facilitate enhanced binding to the active sites of SOD and GSH-Px. Research by Zhang et al. [66] demonstrated that LNT, rich in β-glycosidic bonds, effectively protects against oxidative stress, elevating SOD levels while suppressing MDA content. Additionally, cellular assays demonstrated that LNT exhibits direct free-radical-scavenging activity [67]. Conversely, CMP and LBP potentially ignite Nrf2-mediated ARE-driven transcriptional programs, orchestrating concerted upregulation of catalase at transcriptional and functional tiers [68]. In the liver, all polysaccharide groups significantly upregulated SOD activity, demonstrating a unified mechanism for alleviating hepatic oxidative stress.

Inflammatory and oxidative insults compromise intestinal barrier function, where villus height and crypt architecture constitute sensitive morphological parameters of mucosal integrity [69]. The maintenance of barrier function critically depends on tight junction proteins: Occludin anchors the tight junction (TJ) strand [70], ZO-1 mediates the Occludin–actin linkage for mucosal repair, and MUC2 forms a mucus layer to isolate pathogens [71]. As prebiotics, polysaccharides exhibit gut barrier-protective effects in animals. Chen et al. [46] demonstrated that 600 mg/kg Morus alba polysaccharides attenuate the downregulation of ZO-1 and Occludin mRNA expression in the intestine. Jiao et al. [72] reported that 50 mg/kg Dendrobium huoshanense polysaccharides significantly upregulate ZO-1 and Occludin protein expression in mice. Liu et al. [27] showed that 500 mg/kg black rice polysaccharides (BRP) improve intestinal morphology and upregulate MUC2, ZO-1, and Occludin mRNA expression. At the cellular level, Luo et al. [73] found that 50 μg/mL Bletilla striata polysaccharides enhance ZO-1 and Occludin expression in LPS-treated intestinal epithelial cells, alleviating inflammatory responses. Our results demonstrated that polysaccharides from diverse sources effectively attenuated LPS-induced mucosal damage across small intestinal segments and enhanced jejunal tight junction protein mRNA expression, consistent with prior findings.

The gut microbiota is highly sensitive to external stimuli such as dietary changes and host physiological alterations [74]. Dysbiosis affects not only the gut, but also systemic organs and functions. The intestinal microbiota can regulate immunity and secrete metabolites, etc., mediating inflammatory responses and oxidative damage. The administration of LPS served as a potent stimulus, leading to a marked elevation in the abundance of *Proteobacteria* and *Campylobacterota* within the gut microbiota of mice. Concurrently, it was observed that the levels of *Firmicutes*, which constitute a prominent phylum in the healthy gut microbial community, underwent a significant reduction. *Firmicutes* play a pivotal role in the production of SCFAs. These SCFAs exert their influence by inhibiting the NF-κB signaling pathway through the activation of the GPR43 receptor, as evidenced by numerous studies [75,76,77]. Research by Liang et al. [78] thoroughly demonstrated that the administration of AAP significantly elevated the levels of SCFAs, particularly acetate, propionate, and butyrate, within the intestines of mice with DSS-induced IBD. These findings align with the increased abundance of beneficial bacteria producing these SCFAs observed in the current study. *Campylobacterota* and *Proteobacteria* are Gram-negative bacteria. Their enrichment aggravates inflammatory responses [79,80,81,82]. Alexander et al. [80] demonstrated that Splenda increases *Proteobacteria* abundance, exacerbates inflammatory responses, and elevates myeloperoxidase (MPO) levels—a marker that amplifies oxidative stress. At the genus level, all polysaccharide treatments attenuated the decline in *Lachnospiraceae* abundance and suppressed the enrichment of *Sutterella* and *Helicobacter*, aligning with phylum-level observations. As a member of the Firmicutes, *Lachnospiraceae* degrades complex polysaccharides to enhance nutrient metabolism. Its depletion is linked to gut dysbiosis and intestinal inflammation [33,83,84]. Both *Sutterella* and *Helicobacter* exhibit pro-inflammatory capacities in the gut [85,86]. Their enrichment upregulates inflammation-associated gene expression and elevates MDA levels [86]. Zhou et al. [87] reported a positive correlation between *Sutterella* abundance and pro-inflammatory cytokine secretion. Yu et al. [88] found that the pharmacological reduction of Helicobacter alleviates both inflammation and oxidative stress in mice. Correlation analysis revealed that plasma cytokine levels varied inversely with Lactobacillus density, whereas antioxidant capacity showed direct proportionality. *Akkermansia* abundance was negatively correlated with pro-inflammatory factors, aligning with prior reports [70,89,90].

Natural AAP and BRP exhibit high viscosity [35,91,92]. Their viscous properties enable the formation of a gel-like matrix within the intestinal lumen. This matrix provides dual protection: acting as a physical barrier that reduces intestinal mucosal damage from harmful substances [93], while simultaneously functioning as a prebiotic substrate for beneficial bacterial communities. Dietary supplementation with AAP and BRP significantly enriched *Verrucomicrobiota* in murine models, with *Akkermansiaceae* and the genus *Akkermansia* identified as the primary enriched taxa. Notably, the interaction between *Akkermansia muciniphila* and goblet cells may represent a key mechanism underlying AAP and BRP-mediated intestinal protection. *A. muciniphila* colonizes the intestinal mucus layer, degrading mucin glycoproteins and generating SCFAs. These metabolites provide energy to the host while facilitating bacterial colonization [94,95]. Mucin degradation triggers compensatory mucin production by the host, maintaining a dynamic equilibrium of these glycoproteins [96]. Furthermore, *A. muciniphila* upregulates tight junction protein expression, thereby improving intestinal barrier integrity [97,98]. These mechanistic insights align with our qPCR data. Critically, comparative analyses demonstrate that AAP and BRP exert superior protective effects on the intestinal mucosal barrier relative to other polysaccharides, confirming their enhanced efficacy in mitigating barrier damage.

Notably, although LPS effectively induces intestinal inflammation in mice—manifesting discernible clinical symptoms including weight loss and diarrhea—it primarily elicits acute intestinal inflammatory responses, which differ from the chronic intestinal inflammation characteristic of IBD [99]. In contrast, DSS, currently a predominant IBD-inducing agent, directly destroys small intestinal epithelial cells through chemical toxicity [100], exhibiting pathological features dominated by a Th1/Th2 mixed immune response [101,102]; meanwhile, 2,4,6-trinitrobenzenesulfonic acid (TNBS) functions as a hapten that binds intestinal proteins to activate Th1-type immune responses, with its distinctive advantage residing in simulating the dynamic pathological progression from acute to chronic inflammation [103]. Conversely, LPS specifically binds TLR4 receptors to activate the TLR4 signaling pathway, triggering a dramatic elevation in pro-inflammatory cytokine levels and inducing a cytokine storm; its core pathological manifestation is cytokine-mediated secondary intestinal barrier damage, rather than direct physical destruction [104,105,106]. Although DSS and TNBS are widely acknowledged to more effectively mimic IBD’s chronic inflammation and intestinal epithelial destruction features while activating multiple immune pathways [107,108,109], LPS—as a principal component of Gram-negative bacterial outer membranes—primarily mediates signaling through TLR4 receptor binding and the MyD88/NF-κB pathway, rendering it more suitable for studying bacterial infection-associated acute inflammation while being unable to replicate chronic intestinal inflammation’s comprehensive pathological process fully. Nevertheless, precisely this mechanistic divergence establishes LPS as an optimal model for the comparative evaluation of anti-inflammatory interventions through multiple pathways: within LPS-induced intestinal inflammation, the TLR4 receptor pathway is definitively validated as the primary activated cytokine cascade, where its downregulation significantly correlates with reduced inflammation severity [110,111,112]—entirely consistent with our findings where LBP suppressed TLR4 pathway-related gene expression, subsequently diminishing downstream cytokine release and alleviating inflammatory injury; within the LPS model, which avoids direct physical damage to intestinal cells, ROS generation predominantly stems from cytokine-triggered cascades [113]—thus, when polysaccharides exhibit limited TLR4 pathway suppression (e.g., LNT and CMP groups), elevated antioxidant indicators and reduced MDA levels conclusively demonstrate direct oxidative stress inhibition, unequivocally confirming that these two polysaccharides’ antioxidative mechanisms operate independently of cytokine pathway suppression. Regarding intestinal microbiota regulation, while all three inducers promote detrimental bacterial proliferation and reduce beneficial taxa abundance, TNBS’s routine ethanol enema pretreatment and DSS’s direct chemical action on intestinal epithelium physically interfere with microbiota composition [114,115], whereas intraperitoneal LPS injection minimizes such external confounding factors on gut microecology, ensuring that the observed microbial shifts primarily reflect inflammation-mediated regulatory effects [116,117].

Bioactive polysaccharides have been employed as macromolecular carriers in co-delivery systems for bioactive substances due to their excellent biological activity, in vivo stability, and safety [118,119,120]. These systems aim to enhance drug stability or achieve sustained release. However, current research primarily focuses on the delivery functions of inert polysaccharides like starch [121], failing to synergistically utilize the inherent immunomodulatory and prebiotic properties of bioactive polysaccharides alongside the biological activities of their payloads. Building on the differential mechanisms of five polysaccharides in alleviating enteritis revealed in this study, we propose a “mechanism-guided synergistic combination strategy.” This approach enables synergistic optimization of gut microbiota modulation, immune homeostasis restoration, and oxidative stress mitigation through the precise matching of polysaccharide functionalities. This strategy addresses the intrinsic limitations of combined bioactive agents: Bioactive polyphenols such as curcumin exhibit superior antioxidant capacity (scavenging free radicals and chelating transition metals to inhibit metal-mediated oxidation) [122] compared with polysaccharides, but suffer from poor biostability [123]. Meanwhile, stilbenoids like pinosylvin demonstrate potent antibacterial and anti-inflammatory activities [124,125,126], yet high concentrations may induce oxidative stress, apoptosis, autophagy, and microbiota dysbiosis. Mechanism-guided combinations offer solutions: Combining LBP—which exhibits TLR4 pathway inhibition and prebiotic properties—with curcumin enhances polyphenol stability while synergistically improving TLR4 regulatory function. Alternatively, coordinating AAP/BRP—noted for their prebiotic functions—with pinosylvin enhances biosafety while minimizing disruption to the gut microbiota.

It should be noted that, although this study systematically compared different polysaccharides’ enteritis-alleviating mechanisms, limitations exist. First, the LPS-induced acute enteritis model cannot fully replicate the complex chronic pathogenesis of human IBD, necessitating cautious extrapolation of polysaccharide effects to clinical IBD contexts. Second, the absence of cellular-level investigations precludes the exploration of deeper molecular mechanisms underlying differential polysaccharide regulation. Furthermore, the industrial application of these five bioactive polysaccharides warrants further feasibility studies. Priority should be given to developing AAP and BRP—characterized by high biomass yield and low raw material costs—using water quenching to reduce production expenses. For higher-cost polysaccharides like LBP and CMP, ultrasound-assisted extraction should be adopted to optimize yield [127]. Processing byproducts ought to be repurposed as animal feed to mitigate ecological burdens. Leveraging the complementary mechanisms of polysaccharides (e.g., synergistic effects between LBP-mediated TLR4 pathway suppression and AAP-driven microbiota modulation), formulation strategies can be developed to minimize individual component dosages and costs.

## 5. Conclusions

This study confirms that five bioactive polysaccharides effectively alleviated LPS-induced intestinal inflammation and barrier damage in mice, albeit through distinct core mechanisms: LBP strongly suppressed the TLR4/MyD88 inflammatory pathway; LNT and CMP primarily enhanced antioxidant enzyme activity; whereas AAP and BRP reinforced mucosal barrier function by enriching beneficial bacteria. It should be noted that the LPS model cannot fully replicate the chronic complexity of human IBD. Future research should delve deeper into molecular mechanisms and validate findings in chronic models. These discoveries offer significant theoretical groundwork and application potential for developing functional food ingredients based on complementary mechanisms for targeted intestinal health maintenance.

## Figures and Tables

**Table 1:** Primer sequences of the genes to be tested.

Gene Names	Primer Sequence(5′-3′)
TLR4	F:GCACTGTTCTTCTCCTGCCT
	R:AGAGGTGGTGTAAGCCATGC
MyD88	F:GCATGGTGGTGGTTGTTTCTG
	R:GAATCAGTCGCTTCTGTTGG
NF-κB	F:ACACTGGAAGCACGGATGAC
	R:TGTCTGTGAGTTGCCGGTCT
TNF-α	F:CAAAATTCGAGTGACAAGCC
	R:TTGTCCCTTGAAGAGAACCT
ZO-1	F:GCCTAAAGCTGTTCCTGTGAGTCC
	R:ACCCCGCCGTTGCTGTTAAAC
Occludin	F:TGGCTGCCTTCTGCTTCATTGC
	R:GAACACCATCACACCCAGGATAGC
MUC2	F:CCACAACGACTCCTACGCCATC
	R:CGCTGCCGTCCGACTTGAAG
β-actin	F:AATCCTGCGGCATCCACGAAAC
	R:CAGCACCGTGTTGGCGTAGAG

**Table 2:** Effects of polysaccharides on immune organ indices.(*n* = 6).

Item	Group							*p*-Value
	CON	LPS	LPS+LNT	LPS+CMP	LPS+AAP	LPS+LBP	LPS+BRP	
Spleen index	0.33 ± 0.02 ^b^	0.33 ± 0.02 ^b^	0.49 ± 0.02 ^a^	0.41 ± 0.02	0.50 ± 0.01 ^a^	0.47 ± 0.03 ^a^	0.50 ± 0.01 ^a^	0.003
Thymus index	0.66 ± 0.02 ^Aa^	0.46 ± 0.02 ^Bb^	0.56 ± 0.01	0.58 ± 0.02	0.56 ± 0.01	0.49 ± 0.03 ^Bb^	0.55 ± 0.04	0.003
Liver index	5.62 ± 0.14 ^Aa^	4.08 ± 0.25 ^Cc^	5.35 ± 0.10 ^ABab^	4.86 ± 0.31 ^ABb^	4.73 ± 0.24 ^Bb^	5.21 ± 0.17 ^ABab^	4.92 ± 0.21 ^ABb^	<0.001

Data are presented in this study as the mean ± standard error (SE), (*n* = 6). Within-row values sharing identical lowercase superscripts or no superscripts are statistically equivalent (*p* > 0.05). Distinct lowercase letters denote significant differences (*p* < 0.05), while differing uppercase letters indicate heightened significance (*p* < 0.01).

**Table 3:** The influence of polysaccharides on intestinal morphology (*n* = 6).

Item		Group							*p*-Value
		CON	LPS	LPS+LNT	LPS+CMP	LPS+AAP	LPS+LBP	LPS+BRP	
Duodenum	Velvet length	176.36 ± 16.95 ^Aa^	58.96 ± 8.56 ^De^	99.98 ± 11.58 ^BCcd^	85.78 ± 2.40 ^DCd^	133.56 ± 11.95 ^Bb^	104.65 ± 3.97 ^BCcd^	118.88 ± 6.44 ^BCbc^	<0.001
	Crypt depth	62.98 ± 3.43 ^Aa^	27.41 ± 3.89 ^Dc^	47.37 ± 4.47 ^BCb^	43.12 ± 3.95 ^Cb^	59.54 ± 7.38 ^ABa^	57.66 ± 6.22 ^ABa^	55.67 ± 5.32 ^ABa^	<0.001
Jejunum	Velvet length	154.12 ± 14.11 ^Aa^	53.53 ± 2.12 ^De^	84.87 ± 8.96 ^Ccd^	79.41 ± 4.75 ^Cd^	101.73 ± 3.30 ^Cc^	90.54 ± 14.94 ^Ccd^	124.52 ± 16.62 ^Bb^	<0.001
	Crypt depth	61.71 ± 3.71 ^Aa^	29.74 ± 0.66 ^Cc^	49.04 ± 9.37 ^Bb^	39.48 ± 3.05 ^BCb^	44.53 ± 5.95 ^Bb^	44.42 ± 5.78 ^Bb^	48.82 ± 2.55 ^Bb^	<0.001
Ileum	Velvet length	116.41 ± 6.52 ^Aa^	63.33 ± 3.75 ^Ee^	78.62 ± 4.73 ^Dd^	77.85 ± 8.95 ^Dd^	97.04 ± 1.60 ^BCc^	88.35 ± 1.33 ^CDc^	107.29 ± 7.14 ^ABb^	<0.001
	Crypt depth	69.62 ± 5.88 ^Aa^	31.38 ± 1.06 ^Cc^	43.17 ± 2.98 ^BCb^	44.92 ± 4.73 ^Bb^	45.60 ± 2.38 ^Bb^	41.71 ± 6.18 ^BCb^	44.9175 ± 2.58 ^Bb^	<0.001

The data are presented in this study as the mean ± standard error (SE), (*n* = 6). Within-row values sharing identical lowercase superscripts or no superscripts are statistically equivalent (*p* > 0.05). Distinct lowercase letters denote significant differences (*p* < 0.05), while differing uppercase letters indicate heightened significance (*p* < 0.01).

## Data Availability

The original contributions presented in this study are included in the article, further inquiries can be directed to the corresponding author.
